# The Book World of Medicine and Science

**Published:** 1907-04-13

**Authors:** 


### The Book World of Medicine and Science.

### STRAY NOTES.

**A Foreword.**

When a paper starts on its career, or when it broadens its old road and extends it so as to reach to farther fields, it is permissible for it to give its readers a brief outline of its intentions. This section of The Hospital is, in a sense, a new departure, and one which we trust will be found acceptable to our readers. Its aim is, in brief, to bring to the notice of the practitioner the leading points in the literary history of the week. In the rush of the day's work few of us have time, or inclination, to find out for ourselves what books are worth reading, to discriminate between that volume which gives momentary pleasure and that which makes a man the richer for having read it. We need a guide, or a sign-post to show us the way, and it is desirable that the guide should not be prolix, nor the sign-post overloaded with directions. To that end this section has been designed. It will endeavour to give, weekly, a summary of such books as may reasonably be expected to interest the general practitioner, and of literary articles appearing in the periodicals which may have a bearing on the doctor's work. And in considering what will affect us, as medical men, our range will be catholic and not purely officinal. "Man is more than constitutions," and it will be the aim of this section to deal, succinctly, though of necessity, briefly, not only with purely medical works, but with books which lie outside the circle of professional literature, and which, for that reason, are so often neglected in the scientific press. We appeal to readers to help us in striving to make the section as complete as possible. Suggestions for its improvement, criticisms of its shortcomings, and exposure of its defects will always be welcomed by the editor, and will help to make the section what we desire it to become, namely, a helpful and useful epitome of the week's literary ventures.

**The Medical Aspects of Shakespeare.**

In the current number of the *Dietetic and Hygienic Gazette*, Dr. Wainwright continues his articles on the medical and surgical knowledge of Shakespeare. Someone has said that either in the Bible or in Shakespeare one can find a text for everything, and Dr. Wainwright shows that at any rate the great dramatist can furnish headings for medical essays. Still, we are surprised to find that he only credits his author with four anatomical matters—pia mater, artery, hair, and liver. A Shakespeare student would be able to add a good many others to the list. As proof of the fact that the dramatist knew of the circulation of the blood, Dr. Wainwright quotes Brutus in "Julius Cæsar," and from the fifth scene in the first act of "Hamlet." He might have added Menenius' speech as an even better argument, not of the fact that Shakespeare anticipated Harvey, but that the dramatist had probably read Frascatorius' poems. Many of the quotations adduced to show the dramatist's knowledge of the physiological functions of the body are certainly very striking, and the article is a valuable contribution to the study of the many-sidedness of the author of "Coriolanus."

**Anticipations.**

In "The Strange Case of Dr. Bruno" Dr. Daniel has attempted to write a romance on a particularly gruesome subject. Moreover, he has contrived to imbue his writing with a fascination that will find him many readers—and perhaps a few adherents. His hero is a medical man who is keenly alive to the benefits to be obtained from experimenting on animals, and who has the courage to suggest that vivisection is as legitimate a means of obtaining knowledge when practised on human beings as on animals. Dr. Bruno wishes to experiment on convicted criminals, and deplores the loss which science suffers owing to the "useless execution of condemned murderers." He asks, reasonably enough from his point of view, "why not subject these creatures to experimental studies on the internal organs to solve the problems of immunity, fermentation, and glandular action?" As society refuses to listen to him, he starts experimenting on himself. He studies sleep, and decides to put himself in a trance for six months. Interwoven with all this is his life-story—a tale of miserable, tragic failure and pitiful deception. After his trance he awakes—only to die from the effects of his experiment. The book is not a masterpiece, but it "gives to think," and probably that was the main object its author had in view when he penned it. It is published by the Guarantee Publishing Company, New York.

**Some Magazines of the Month.**

The April magazines do not offer much that is of medical, or even semi-medical interest. But there are a few noteworthy exceptions. In *Chambers's Journal* an anonymous writer discusses "Doctors, Old and New," in a chatty and informative article, which well repays its reading. It bristles with anecdotes, many of which will, however, be well known to most medical men. One citation of a story which we do not recollect having seen in a popular magazine, must suffice. "Caesar Hawkins, when in company with Robert Lee, who had kicked a bit of orange-peel from the pavement to the roadway, replaced it with the words, 'What are you thinking about?'" This sly hit at the expense of the profession was originally told at the expense of Cheselden. There are a few errors of fact in the article, but on the whole it is an extremely well-written anecdotal sketch of old and new medicos. In the *Royal Magazine* a K.C. describes his day's work, which appears to be almost, if not quite, as laborious as that of a general practitioner. In the *English Illustrated* Mr. Sidney Hunt gives an interesting pen-picture of some old English homes, most of them associated with historical events. The *Fortnightly* is an unusually interesting number. Professor Turner's article on "Man's Place in the Universe" is an able contribution to the discussion initiated by Dr. Wallace, and Mr. Minchin's study of Fielding will appeal to lovers of "Tom Jones." The *Grand* has three items which demand notice. One is Mr. Thomas' three-verse narration of how he cheated the doctor by waking the latter in the middle of the night to attend what he made out to be an urgent case at a far-off cottage. The doctor went in his carriage, only to find that he had been hoaxed, for the story ends as follows :—

> Here's your ten and sixpence, doctor,
> It's a good long way from town—
> And I couldn't find a hansom
> Under twenty shillings down."

Dr. Bell writes on "A Medical Conundrum," and pleads vigorously for fresh air and less coddling. Dr. Sebroff contributes a paper on "Delusions," describing some interesting cases he has met with. The *Badminton* has two informative articles on out-of-the-way hunting grounds—stag-hunting on the Campagna, and wild-fowling in Burma. *C. B. Fry's* devotes several pages to "Hints on Housing a Cycle," from which the cyclist may derive much useful information, and further on gives a paper on "Points to Study in Choosing a Motor-car Body," which the intending motorist will do well to study. In the same number is an interesting article on old Inter-University boat crews. From it we learn that by far the majority of them became clergymen or lawyers: Only two of the Cambridge men became doctors, while but four of the Oxford blues finally landed in medical practice.

BOOK WORLD—*continued.*

### A LIBRARY OF PROBLEMS.*

Some time ago Messrs. Methuen and Company started, under the general editorship of Dr. C. Saleeby, the publication of a series of volumes entitled "The New Library of Medicine." These volumes were planned "on the assumption that there are certain matters of the very gravest importance which urgently claim the attention and appreciation not only of the medical man, but also of the layman." Written on that assumption—one which we are of opinion is perfectly warranted by facts—these works, of which the two cited above are the most recent, form essentially a library of problems—problems that should appeal to everyone, no matter whether layman or practitioner. Broad questions of vital importance are the subjects with which they deal, and the men who are responsible for them are authorities whose opinions, whether acceptable or otherwise, are at least worthy of thoughtful respect. In a sense, the aim of the originators of the series is a very high one. It is to bring home to the great mass of the reading public the grave aspects of certain subjects which have a marked relation to our personal and national life. Primarily, therefore, it purports to be an educative series. It is designed to instruct and to enlighten, and bearing this in mind, one can overlook a certain amount of dogmatic *ex-cathedra* speaking which would otherwise jar upon the reader. Of necessity such a series must be controversial in many respects, but a careful scrutiny of the volumes which have so far been issued, leads one to the conclusion that the several writers have, as much as possible, stifled the personal note in an honest attempt to throw light on the problems with which they deal. The volumes are uniform in size and shape, clearly printed, and neatly bound in red cloth. Each has a respectable look, such as, indeed, we are accustomed to look for in the works which emanate from the well-known Essex Street publishing house. Issued at the moderate price of 7s. 6d. each net, they are well within the reach of everyone, and offer a variety of subjects from which everyone can choose to his own satisfaction. The volumes which have already appeared are "Infant Mortality," by Dr. Newham; "The Hygiene of Mind," an eminently thoughtful work from the pen of Dr. Clouston, of Edinburgh; "The Children of the Nation," in which Sir John Gorst takes for his text the danger of neglecting the health of the nation's children; and the two volumes with which we here deal more fully. In preparation are "The Care of the Body," "Diseases of Occupation," "The Hygienics of Education," "The Prevention of Tuberculosis," "Nutrition," "Drugs and Drug Habits," "Air and Health," "Functional Nerve Diseases," "Mentally Defective Children," "Serum Therapy," "The Insane," "Heredity," "Infection," and "Imperial Hygiene." The list shows how widely Dr. Saleeby has interpreted the meaning of "personal and national importance," and how exhaustively he has attempted to deal with the main subjects. In "The Control of a Scourge" Mr. Childe sets himself the difficult task of dealing with the problem of cancer. After reviewing, in a style which is perhaps more popular than strictly scientific, the many theories concerning the disease, he goes on to deal with the clinical aspects of cancer, and finally to point out lucidly the lines upon which it may be combated. In an interesting chapter he shows the failure of the many so-called cures—Christian science, *x*-rays, high-frequency currents, cancroin, violet leaves, molasses, trypsin, Otto Schmidt's method, and the host of other means of treatment which have been advocated. Practically, he pins his faith to the teaching that early and radical operation is the only hope of eliminating the disease. In this he will be supported by every thinking man who has had even a moderate experience of cancer cases. His whole book is an eloquent appeal for the education of the laity—an appeal that The Hospital, on more than one occasion, has voiced in no half-hearted fashion. By teaching the public the danger signals, by showing them that their only hope of relief is to seek the surgeon's aid at the first sign of the disease, and by overcoming their morbid dread of operation except as a last resource—it is by these paths, Mr. Childe points out, that the ultimate goal of success may be reached. To such suggestions some of us will reply that inadequate instruction in medical matters is worse than none. There is no quotation more often misapplied than "Drink deep or taste not," and for the arguments against it in reference to this subject, the reader may safely be referred to Mr. Childe himself, whose earnest and temperate *résumé* of the pros and cons of the case is eminently worthy of an equally earnest and temperate consideration.

More debateable, because involving broader issues, is "The Drink Problem," in which fourteen medical authorities discuss the medico-legal and medico-sociological aspects of alcoholism. There are many points, in reading this book, with which we find ourselves in agreement with the writers, but there are as many upon which we must join issue with them. The purely scientific chapters—among which Professor Sims Woodhead's synopsis of the pathology of alcoholism, and Dr. Hyslop's account of alcoholism and mental disease, are worthy of special mention—may be dismissed with a few words. They are instructive contributions to the medical literature of a subject which has already been fully treated. But the more general chapters demand a word of further comment. Most of us will be fully with Dr. Crowley in ascribing the large increase in pauperism to alcoholism, but how many will cordially agree with Dr. Jones that intoxicating drink is the main factor in national deterioration? The optimist may be pardoned for questioning if national deterioration has been proved, at any rate in a strictly scientific sense. We want more statistics, more facts, and more figures before an adequate reply can be given. So, too, when we come to the alleged increase in the consumption of alcoholic liquors, it appears to us that the authors have not made out a clear case that there is a definite increase. That alcohol is a factor in the evolution of the criminal we are all prepared to admit; that it is a daily increasing factor in causing a break-down in women, as Mrs. Scharlieb would have us believe, we may also admit. But that alcoholism is a greater factor in the evolution of the criminal to-day than it was fifty years ago, and that the economic evolution of the woman worker has tended to produce more intemperance, are propositions to which we are by no means prepared to subscribe. In the final chapter, Dr. Kelynack deals with the "Arrest of Alcoholism," and suggests measures for dealing with the problem. These we do not propose to discuss, beyond saying that they appear to us to stop short of the radical measures proposed by Dr. Archdall Reid in his well-known book published some years ago.

The thoughtful reader will find much food for reflection in both these volumes, and the very fact that both of them are open to discussion makes them more interesting, and therefore more valuable. Both are short, easily read, and, to the medical practitioner at least, both should prove of service.

* "The Control of a Scourge." By Charles P. Childe, B.A., F.R.C.S.
"The Drink Problem in its Medico-sociological Aspects." By Fourteen Medical Authorities. Edited by T. Kelynack, M.D., M.R.C.P. Methuen and Co. 7s. 6d. each net.

